# Recovery of *Sphagnum* from drought is controlled by species-specific moisture thresholds

**DOI:** 10.1038/s41598-025-05348-8

**Published:** 2025-07-01

**Authors:** Ben Keane, Emma L. Shuttleworth, Martin G. Evans, Jonathan P. Ritson, Angela Harris, Adam Johnston, Danielle M. Alderson, Gareth D. Clay

**Affiliations:** 1 School of Environment, Education and Development, The University of Manchester, Oxford Rd, Manchester, M13 9PL UK; 2 Department of Environment and Geography, University of York, York, YO10 5NG UK; 3 Department of Geography, Durham University, DH1 3LE Durham, UK; 4 JBA Consulting, 1 Broughton Park, Old Lane North, Broughton, Skipton, North Yorkshire, BD23 3FD UK

**Keywords:** *Sphagnum*, Drought, Recovery, Methane, Carbon, Peatland, Ecology, Biogeochemistry, Climate sciences, Environmental sciences

## Abstract

As the largest terrestrial carbon (C) store, peatlands are vital to meeting climate targets. *Sphagnum*, a genus of ca. 350 species, sustains many peatlands through its high water content and chemistry which inhibits decomposition and vascular plant proliferation. However, many peatlands face increased risk of drought due to climate change, and how *Sphagnum* will respond and recover from drought is unknown. We measured moisture content, CO_2_ and methane (CH_4_) flux, and photosynthetic pigments in two species, *S. palustre* and *S. squarrosum*, over increasing drought (1–10 weeks) and recovery (1–10 weeks) periods. We identified biomass moisture thresholds of 12 g g^− 1^ (*S. palustre*) and 18 g g^− 1^ (*S. squarrosum*) below which irreversible damage occurred to photosynthesis. Due to higher moisture retention, and a lower moisture threshold, *S. palustre* withstood longer drought than *S. squarrosum*. These species-specific thresholds provide important insight for modelling peatland C sinks and for sustainable peatland restoration.

**Supplementary Information:**

The online version contains supplementary material available at 10.1038/s41598-025-05348-8.

## Introduction

Anthropogenic climate change is on course to heat the planet beyond the agreed “safe” target of 1.5 °C^1^, driven by atmospheric greenhouse gases (GHGs) including carbon dioxide (CO_2_) and methane (CH_4_). The magnitude of historic and ongoing GHG emissions means that reducing future emissions alone will be insufficient to minimise the harmful effects of climate change. We now require effective ways to actively remove GHGs from the atmosphere, including natural climate solutions^2,3^. Terrestrial ecosystems have the potential to be one such solution, absorbing 112–169 Pg carbon (C) per year^4^, but this terrestrial sink itself is vulnerable to climate change: in 2023 it has been estimated that this sink nearly shut down entirely^5^. As the largest terrestrial store of C, peatlands are perhaps the most important biome in this context^6^, storing 500 Gt C globally and on average accumulating 23 g C m^− 2^ y− 1^7,8^ in wet environments which prevent dead organic material decomposing.

However, human management has led to a loss of ecological function in many peatlands, with as much as 80% of these ecosystems being described as ‘degraded’ across the United Kingdom (UK)^9^ Europe, North America and every continent except Antarctica. As vast stores of C, peatlands also represent potential sources of CO_2_ should they dry out, either deliberately through drainage, or by a changing climate. Climate projections indicate that rainfall patterns will alter in latitudes where peatlands are prevalent, leading to more frequent, prolonged periods of drought^10^ interspersed with intense rainfall events. In the UK alone, desiccation events in peatlands are predicted to increase by 44–82% by 2080^11^. The effects of drought on peatlands include increased greenhouse gas emissions, especially CO_2_ and CH_4_^12^, biome shift^13^ and increased risk of wildfire^14^. Globally peatlands also deliver multiple non-carbon ecosystem services (ES), such as hydrological regulation^15^cultural and recreational^16^ and agriculture^17^. To manage these vital global ecosystems to be resilient to future climate, and to sustain delivery of their key ES, we must understand how they respond to and recover from drought.

*Sphagnum* mosses are key to non-tropical peatland functioning, and specifically C cycling^18,19^. They may cover 80–100% of peatland land area^20^ and it has been estimated that C in *Sphagnum* biomass exceeds that of any other land plant genus^21^. Whilst the rate of gross primary productivity (GPP, photosynthesis) of *Sphagnum* may not be as great as vascular plants, *Sphagnum* gains a competitive advantage through using nutrients much more efficiently^22^. Thus, *Sphagnum* inhibits growth of vascular plants while creating conditions favourable to its own productivity^21^; it holds many times its own weight in water, maintaining the wet conditions vital to sustain peatlands^23^; the chemistry of *Sphagnum* necromass slows decomposition, leading to the build-up of organic material which forms peat^22^. The specific niche of individual species varies, for example along a hydrological gradient between microtopographical features in the landscape, with some species growing as lawns in wet hollows and others forming hummocks further from the water table^24^. Further, *Sphagnum* has also been shown to host methanotrophic microorganisms, which reduce the net emissions of CH_4_ from wetlands^25^. Hence, much of the focus of peatland restoration projects centres on *Sphagnum* reestablishment^26^.

The response of *Sphagnum* to drought and rewetting will be key to peatland functioning under future climate, but it is not well understood. There are *Sphagnum* drought studies covering periods from a few hours^27,28^ to weeks^23,28–30^ and they tend to focus on a narrow range of *Sphagnum* species^31^. Investigations of *Sphagnum* recovery are less common and vary in the detail studied. Rewetting periods may just be a few hours^32–34^ days^27,35^ or weeks^36–38^ and the most common way to monitor has been to compare GPP to pre-drought levels. The challenge to interpret these studies is the variation between drought recovery periods, which are often arbitrary.

Our objective in this study was to subject *Sphagnum* to increasing drought and rewetting periods to determine the threshold, or ‘breaking point’, beyond which it will not recover. We chose to use two under-studied but globally relevant *Sphagnum* species, closely related phylogenetically, but with different microhabitat preference: *S. palustre* is a hummock forming species and *S. squarrosum* an intermediate, often forming lawns in wetter areas alongside *S. palustre*^39^. The two species are extremely common throughout Europe, the Americas and Asia and Oceania (Atlas of British & Irish Bryophytes). Due to their niche differentiation, we hypothesised that hummock forming *S. palustre* would withstand longer drought periods due to its adaptation to growing further from the water table than *S. squarrosum*. We measured moisture content and C cycling by measuring net ecosystem exchange (NEE) of CO_2_ and CH_4_ from *Sphagnum* subjected to increasing drought periods from one to ten weeks and after periods of rewetting from one to ten weeks. At the end of the experiment, we also collected hyperspectral data to calculate key vegetation indices linked to moisture and photosynthetic pigments^40,41^ from all droughted and rewetted treatments and compared them to our controls. This not only allowed non-destructive investigation of the effect of drought on *Sphagnum* photosynthetic apparatus, but provided key information for remote sensing drought damage for peatlands.

## Results

In the following sections covering *Sphagnum* water content, NEE of CO_2_ and CH_4_ fluxes, the statistical differences between species, drought treatment and time, are inferred from the results of linear mixed effects models which included species, drought treatment and time as fixed effects and experimental block as a random effect (please see Methods).

### *Sphagnum* water content

**Fig. 1:**
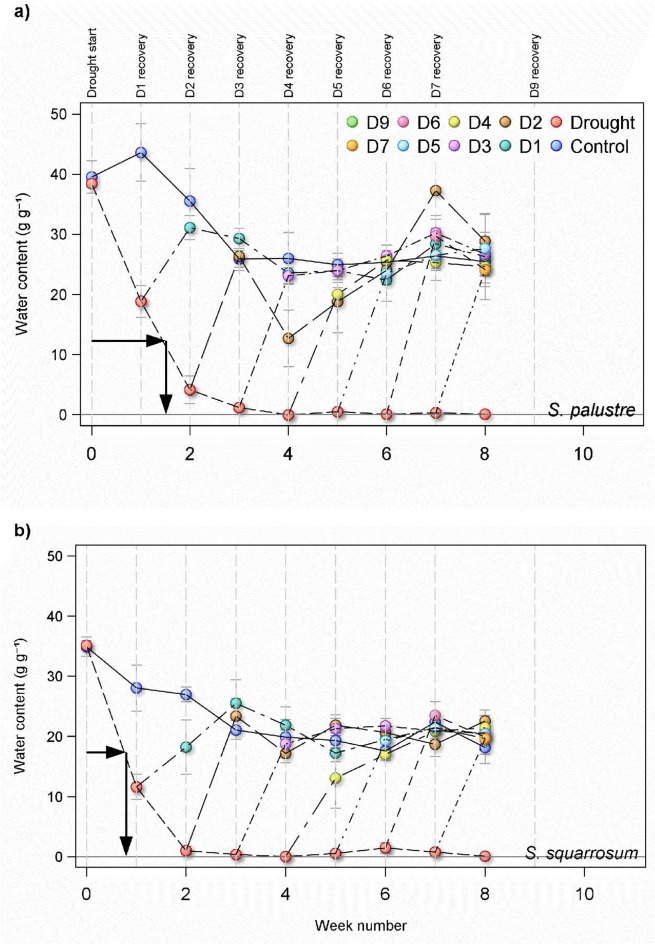
Water content of *Sphagnum* biomass from microcosms of two species (*S. palustre* (a) and *S. squarrosum* (b)). Moisture content is expressed as the ratio of water content (mass, g) to the dry biomass of *Sphagnum* (g). From week 0 onwards, *Sphagnum* was either kept inundated with rainwater (100 ml weekly, control (blue symbols)), droughted (removal of water and nothing added, drought (red symbols)) or rewetted. Data shown are means for each treatment (*n* = 5 ± 1SE, with the exception of Drought, where initial *n* = 45 and declined by 5 each week). The horizontal arrows indicate the optimum moisture level for NEE in each species (see later section), and the vertical arrows indicate the timepoint at which the biomass dropped below this level.

Moisture content differed significantly between the two *Sphagnum* species (*p* < 0.0001), with the undroughted controls of *S. palustre* holding 37.2 ± 2.5 g g^− 1^ compared to 28.0 ± 2.5 g g^− 1^ in *S. squarrosum*, representing a difference of 32% in moisture retention (Fig. 1). This difference was seen across all the drought treatments (*p* < 0.0001), where the average water content across all treatments was 20.7 ± 1.5 in *S. palustre* versus 16.2 ± 1.5 in *S. squarrosum*.

Once excess water was removed from the drought microcosms, moisture content declined rapidly in both species (Fig. 1), though the rate of decline was slower in *S. palustre*, which retained a moisture content of ca. 20 g g^− 1^ after the first week of drought, compared to ca. 12 g g^− 1^ in *S. squarrosum*. After two weeks of drought this dropped further, to 5 g g^− 1^ in *S. palustre*, compared to 2 g g^− 1^ in *S. squarrosum*. By the third week of drought *S. palustre* had dropped to 2 g g^− 1^ and *S. squarrosum* to 1 g g^− 1^.

Moisture content did vary over time, even in the inundated controls of both species (Fig. 1); there was a slight increase in *S. palustre* after the first week, from 40.5 g g^− 1^ to 44.6 g g^− 1^, before declining to 36.5 g g^− 1^ after week two, to 27.0 g g^− 1^ after week three the difference between sequential weeks was not significant, the overall decrease from start to end of week 3 was significant (*p* < 0.03) in *S. palustre*. Likewise, there was a significant general trend of declining moisture in *S. squarrosum* controls, from 35.9 at the beginning of the experiment to 22.0 g g^− 1^ by the end of week three (*p* < 0.03).

Recovery of moisture content was, for the most part, rapid in both species. In *S. palustre*, D1 and D2 reverted to the same moisture content as controls one week after being rewetted. A similar pattern was seen in *S. squarrosum*, with the exception that D1 took two weeks to recover to the same moisture content as the controls (Fig. 1).

### NEE of carbon dioxide

#### Effect of species

**Fig. 2:**
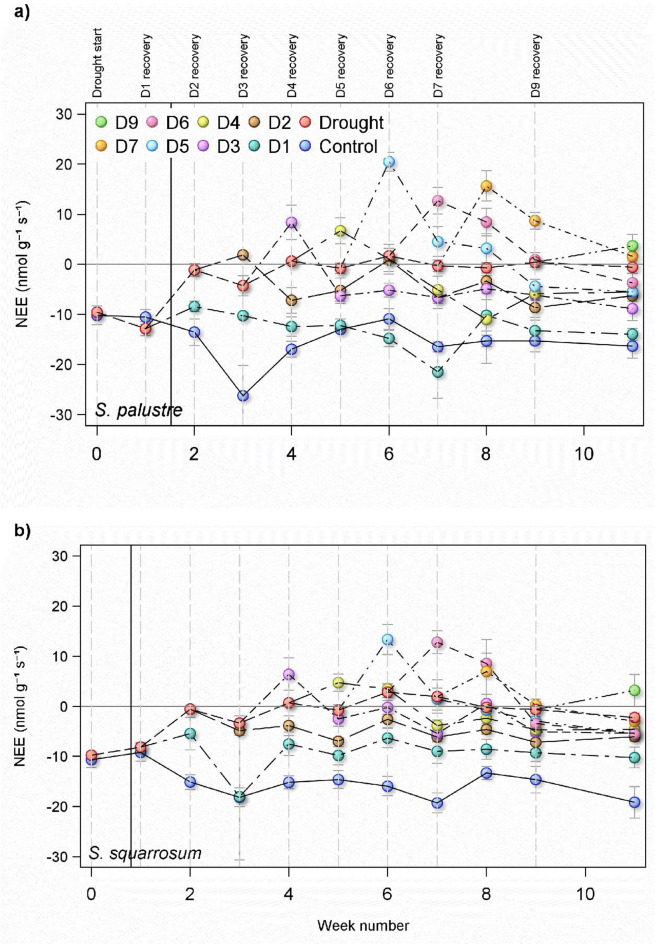
Net ecosystem exchange of CO_2_ (NEE) measured from microcosms of two *Sphagnum* species (*S. palustre* (a) and *S. squarrosum* (b)). Microcosms were kept inundated (control), droughted (drought) or droughted then rewetted after a number of weeks (e.g. D1 = rewetted after 1 week drought). Negative values of NEE indicate net uptake of CO_2_. Vertical dashed lines indicate timing of drought and recovery periods. The solid vertical lines indicate the timepoint at which the *Sphagnum* biomass dropped below the optimum moisture content for NEE, and subsequently failed to recover. Data shown are means for each treatment (*n* = 5 ± 1SE, with the exception of Drought, where initial *n* = 45 and declined by 5 each week).

Both species were net sinks for CO_2_, but NEE significantly differed between species across all treatments (*p* = 0.025), with *S. squarrosum* taking up ca. 22% less CO_2_ than *S. palustre* (Fig. 2), with mean fluxes of − 6.27 nmol g^− 1^ s^− 1^ and − 5.13 nmol g^− 1^ s^− 1^ respectively.

#### Effect of drought treatment

There was a strong significant effect of drought treatment on CO_2_ fluxes across the study (*p* < 0.0001), which differed over time (significant interaction between drought treatment and time, *p* < 0.0001). After one week of drought, fluxes between the controls and drought treatments were the same in both species. It was only by the end of week two that drought took an effect, when CO_2_ fluxes approached zero, and remained relatively steady for the duration of the study. In contrast, the controls of both species saw a general increase in net uptake (fluxes became more negative) across the study. Due to analyser malfunction in week 3, insufficient replicates were measured for statistical testing and therefore these data were removed from the analysis.

#### Recovery from drought

NEE recovered more quickly in *S. palustre* than in *S. squarrosum* (*p* < 0.001; Fig. 2), shown by the similarity in fluxes between controls and D1 in *S. palustre*: at no point in the study did the fluxes differ between controls and D1 treatment. In contrast, in *S. squarrosum* NEE was significantly less in D1 relative to controls at week 2 (*p* < 0.001), and even after three weeks of recovery at week 4, NEE was still significantly lower compared to controls (*p* = 0.017).

When the drought period exceeded one week, recovery was slower in both species. In treatments D2, D3 and D4, fluxes became more positive than the droughted treatments after the first week of rewetting (Fig. 2). In all treatments except D2 in *S. squarrosum*, the microcosms became net sources of CO_2_ after the first week of recovery, after which they reverted to being net sinks once more. At the end of week 5, D2 and D3 were statistically similar, on a trajectory to recover to the same rate as the controls. A similar pattern can be seen in *S. squarrosum*, although the magnitude of recovery in D3 is much less than in *S. palustre*.

### Methane

#### Effect of species

There was a difference in mean CH_4_ fluxes (*p* < 0.06) between the two *Sphagnum* species across the whole study, with uptake greater in *S. palustre* compared to *S. squarrosum* but this did not meet the 0.05 threshold as our other findings. This was largely driven by an initial difference of nearly 100% (*p* < 0.003), with uptake of -3.56 pmol g^− 1^ s^− 1^ in *S. squarrosum*, but − 7.10 pmol g^− 1^ s^− 1^ in *S. palustre* (Fig. S1). However, after the first week there were no differences in fluxes between the two species. What did occur is that fluxes gradually tended towards zero across the study period.

#### Effect of drought

There was no overall effect of the drought treatment on methane fluxes (*p* > 0.1), although in both species there was an initially tendency for uptake to increase in the rewetted treatments, e.g. in D1 at week 2, but this was not a significant effect.

By far the biggest driver of change in CH_4_ fluxes was time (*p* < 0.0001), with fluxes at the start of the experiment (weeks 0 to 2) showing significantly more uptake than later weeks.

### Length of drought and effect on recovery

**Fig. 3:**
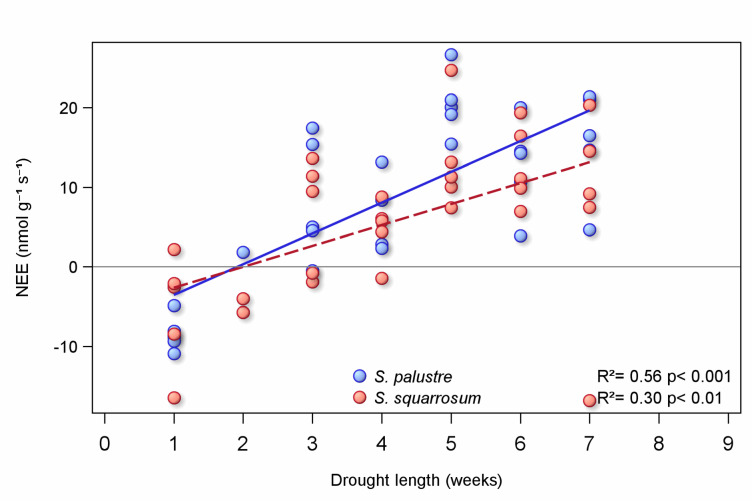
Relationship between length of drought period and fluxes of CO_2_ from two *Sphagnum* species (*S. palustre*, blue symbols and regression line, and *S. squarrosum*, red symbols and regression line), one week after rewetting. CO_2_ fluxes are net ecosystem exchange (NEE). With negative flux indicating sink of CO_2_ from the air and positive fluxes release.

Fluxes of CO_2_ for both species initially became more positive after rewetting (Fig. 2), but this response increased with the length of drought the *Sphagnum* was exposed to (Fig. 3). *Sphagnum* that experienced just one week of drought still showed net uptake one week after rewetting, but after a five-week period of drought followed by rewetting *S. palustre* was releasing nearly 20 nmol g^− 1^ s^− 1^, and *S. squarrosum* ca. 10 nmol CO_2_ g^− 1^ s^− 1^. The rate of increase differed between the two species, with the effect of drought length stronger in *S. palustre* (steeper gradient, Fig. 3).

The rate at which NEE recovered in droughted *Sphagnum* also differed between species. The relative rate of CO_2_ flux is expressed in Fig. S3 as the absolute difference in NEE between each microcosm and the NEE from the control microcosm in each experimental block. Across all drought lengths in both species NEE recovered rapidly following rewetting and levelled off after ca. 7 weeks in *S. palustre*, later in *S. squarrosum* (Fig. S3). The asymptote in both species was well above the rate of NEE in the controls, with final flux rates of 3.03 and 4.25 nmol g^− 1^ s^− 1^ in *S. palustre* and *S. squarrosum* respectively. This indicates that neither species would recover its full C sink properties relative to the controls for a period of months.

**Fig. 4:**
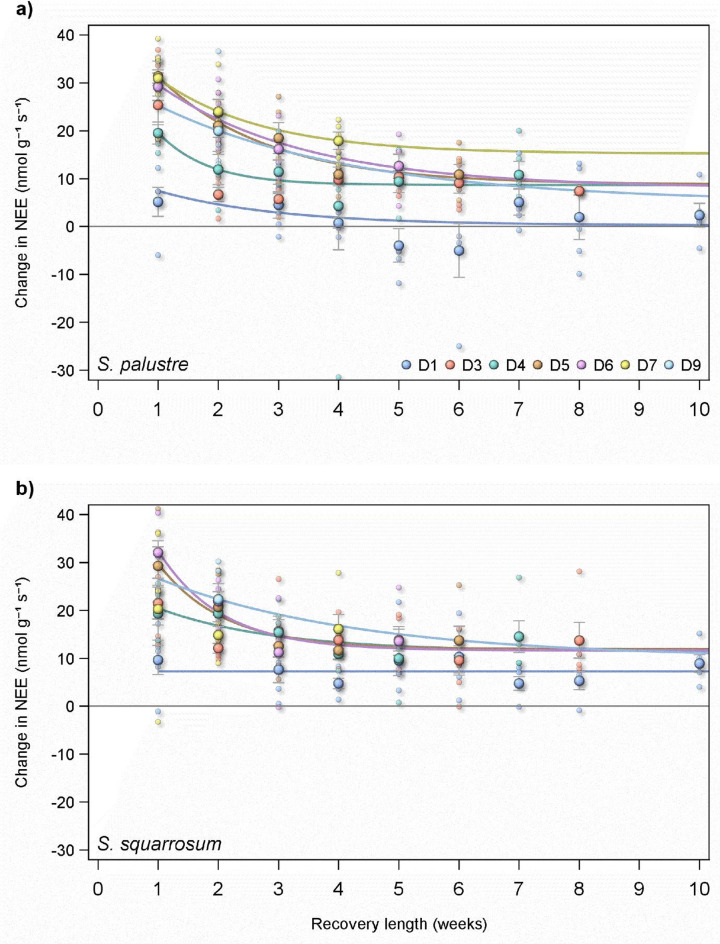
Variation in relative CO_2_ flux (difference in NEE between recovering microcosms and control microcosms) from two *Sphagnum* species (*S. palustre*, (a), and *S. squarrosum*, (b) following rewetting after drought. Within each species, the recovery from different length of drought is indicated by coloured symbols and regression lines (blue = D1, red = D3, green = D4, brown = D5). Regressions follow the same formula as Fig. S3; y = initial flux * e^x^ + final flux). The zero line represents the rate of NEE in controls; fluxes above this (positive fluxes) indicate increased loss of CO_2_ from *Sphagnum* and fluxes below (negative fluxes) indicate increase uptake of CO_2_. Fluxes are means ± 1SD and the regression line fitted is a negative exponential, formulae in the top right of the panel.

However, it is also clear that the length of drought affects both the speed of recovery and the magnitude of recovery of the C sink. In *S. palustre* that has been subjected to a drought of one week, NEE will recover to rates similar to controls 5 weeks after rewetting (Fig. 4). A drought longer than one week leads to a steady state of NEE that is approximately 10 nmol g^− 1^ s^− 1^ greater a source than the controls (Fig. 4). In contrast to *S. palustre*, the sink nature of *S. squarrosum* does not recover to the rates of the controls even after a drought of just one week (Fig. 4).

### Partitioning of C fluxes after rewetting

The observed release of CO_2_ after one week of recovery was measured as NEE, which is the sum of sources and sinks of CO_2_. To investigate whether this release was due to **(i)** Inhibition of photosynthesis (GPP), **(ii)** Stimulation of respiration, **(iii)** Neither / both, CO_2_ fluxes were partitioned by following measurement of NEE by darkening the microcosm and measuring respiration. This was done from week 7 on treatment D6 and the controls. In the controls, there was virtually no dark respiration measured at all, and that NEE was completely driven by GPP. Conversely, in the recovering microcosms, the release of CO_2_ was driven almost exclusively by respiration, and that no photosynthesis was occurring whatsoever; over time, NEE became more negative in the recovering mesocosms, and this was driven predominantly by a decline in dark respiration, with a slight increase in GPP in *S. squarrosum* (Fig. S4).

### Relationship of NEE to biomass moisture

**Fig. 5:**
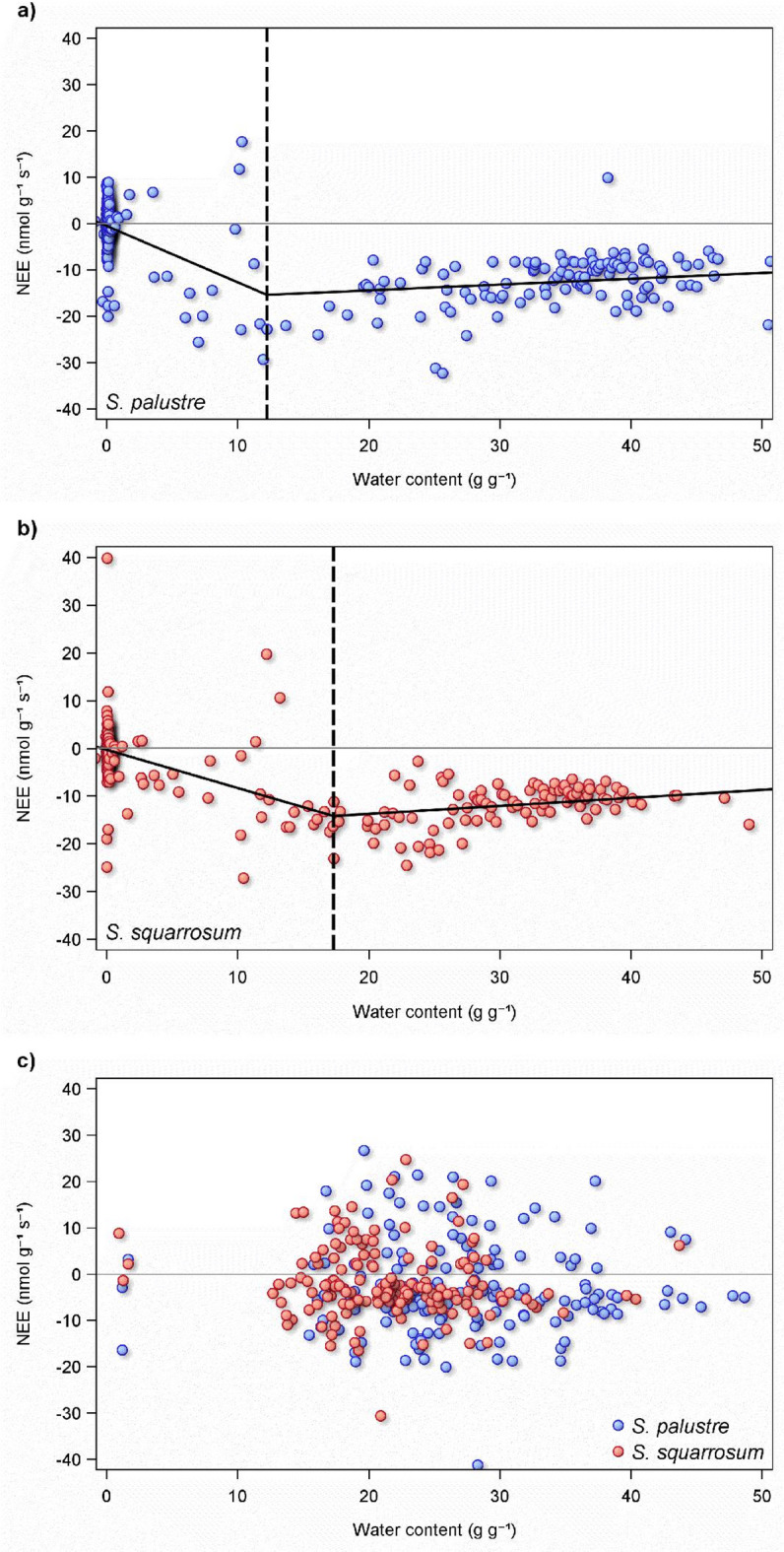
Relationship between biomass moisture content and net ecosystem exchange (NEE) of two *Sphagnum* species before drought and after: (**a**) (*S. palustre*) and (**b**) (*S. squarrosum*) panels show the relationship between moisture content and NEE from controls and droughted microcosms during the drying phase of the experiment. Segmented regression models indicate that there is an asymmetric relationship, with an optimum moisture content for both species (dashed vertical line), above and below which NEE declines, but at different rates. This point is different for each species. (**c**) Shows both species after rewetting, and how the relationship between moisture and NEE breaks down after drought.

There was a clear hysteresis between *Sphagnum* moisture content and NEE, delineated by pre-drought and rewetting (Fig. 5). Prior to and up to drought, there was an asymmetrical relationship between moisture content and NEE around an optimum level of moisture. This optimum was higher in *S. squarrosum* (ca. 18 g g^− 1^) than in *S. palustre* (12 g g^− 1^). The asymmetry was characterised by a slow decline in NEE above the optimum level of moisture (i.e. NEE becoming less negative), but a steeper rate of decline below the optimum level of moisture. After rewetting, however, this relationship collapsed entirely in both *Sphagnum* spp. (Fig. 5, bottom panel).

### Hyperspectral reflectance

The bleaching incurred by continuous drought was evident from the high level of reflectance across all wavelengths (Fig. S5). There was also separation between the drought periods, which is particularly evident in various vegetation indices.

There was a significant effect of drought length on all indices measured (Figure S6): NDVI (*p* < 0.0001), CRI (*p* < 0.0001), PSRI (*p* < 0.0001), CAI (*p* < 0.01), ARI, (*p* < 0.0001), PRI (*p* < 0.0001), SIPI (*p* < 0.0001) and REP (*p* < 0.0001); with the exception of CAI, these showed a general decline in index from controls (highest) to continuous drought (lowest); REP increased significantly with drought length.

Of particular interest is the interaction between species and drought length in CRI (*p* < 0.03). This index is higher in *S. palustre* under control and 1 week drought, but flips to be substantially higher in *S. squarrosum* after two weeks drought. This reflects *S. palustre*’s ability to withstand drought for longer than *S. squarrosum*: in *S. palustre* there is a significant decline in CRI between D1 and D2, but in *S. squarrosum* these treatments are similar, and the decline occurs at 1 week (i.e. D1 and D2 are statistically the same in *S. squarrosum*).

CAI also showed significant effects of species (*p* < 0.02) and an interaction between species and treatment (*p* < 0.001). In this instance, rather than a decline with drought length, there appeared to be very little variation between the controls and the rewetted *Sphagnum* across both species. There was a very large difference in how the continuous drought affected the species; increasing CAI in *S. squarrosum* but no change was observed in *S. palustre*.

**Fig. 6:**
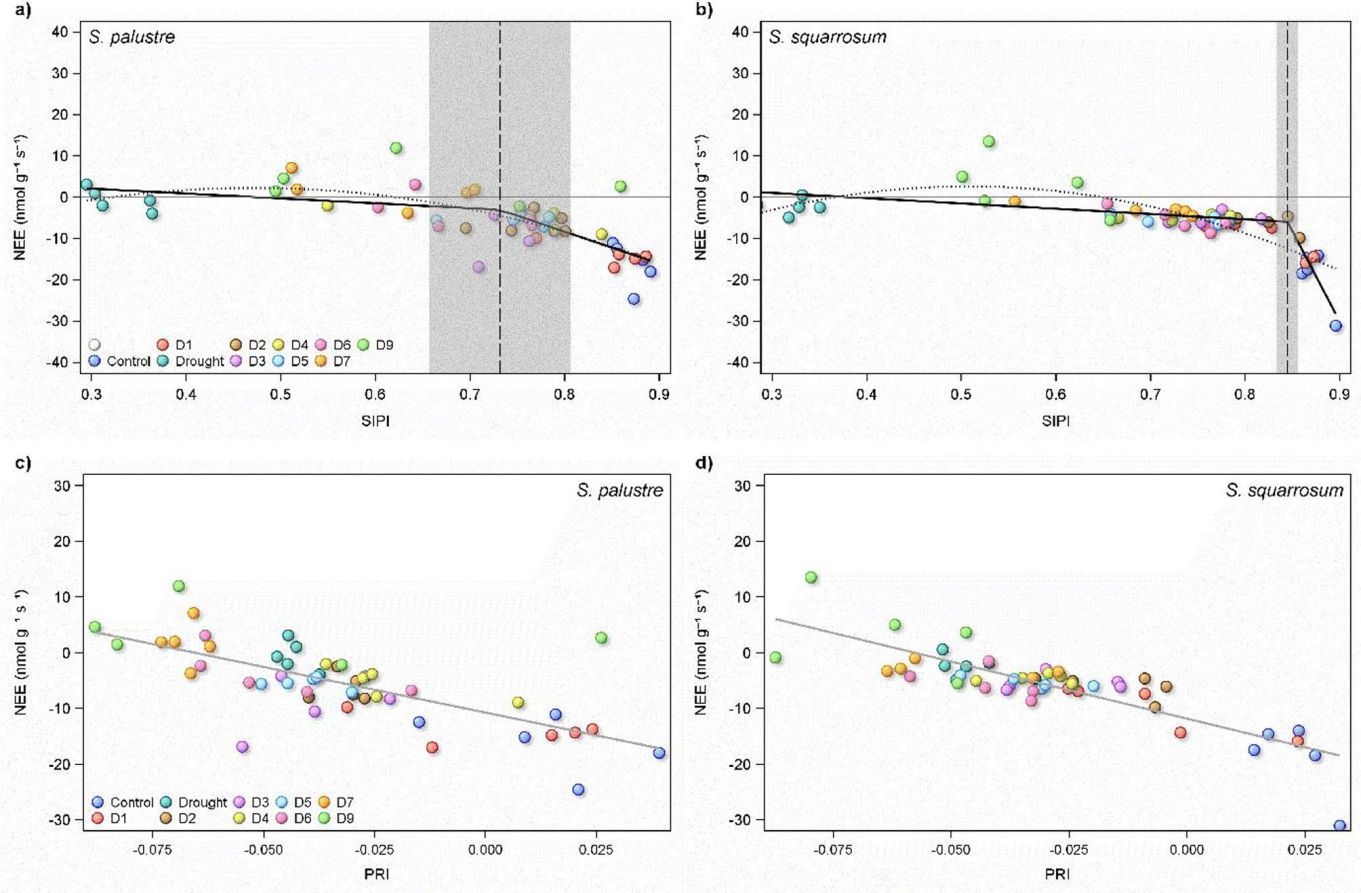
Relationship between structure insensitive pigment index (SIPI) and NEE in *S. palustre* (**a**) and *S. squarrosum* (**b**) under different levels of drought and recovery (Control, constant drought (Drought) and rewetted after one week drought (D1) up to nine weeks drought (D9). Dotted regression line indicates a second derivative polynomial (quadratic) fit. Solid black line indicates segmented linear regression, with the break point illustrated by the vertical dashed line. The 95% confidence intervals of the breakpoint are demarcated by the vertical grey band. Relationship between photochemical reflectance index (PRI) and NEE in *S. palustre* (**c**) and *S. squarrosum* (**d**). The grey solid line indicates the linear regression.

Flux of CO_2_ was best described in *S. palustre* by SIPI, which demonstrated a quadratic relationship (Fig. 6a, R^2^ = 0.56). While there was also a significant relationship between SIPI and NEE in *S. squarrosum* (Fig. 6b, R^2^ = 0.66), it was not the best predictor of fluxes in this species. Further, there appeared to be a distinct break point in the influence of SIPI on NEE in *S. squarrosum* (Fig. 6, bottom panel), which was much less clear in *S. palustre*, reflected in the large variation in the range of the confidence intervals around the break points in the two species (Fig. 6). The best predictor of NEE in *S. squarrosum*, was PRI, which displayed a linear relationship in both species (Fig. 6d *S. squarrosum* R^2^ = 0.70, Fig. 6c *S. palustre* R^2^ = 0.49), with no indication of a threshold or break point in the relationship.

## Discussion

The ca. 350 species within the *Sphagnum* genus largely differentiate between the two microforms of hummock and hollow^24^and the model species selected for our study not only represent each of these microforms, but are represented on multiple continents throughout the global distribution of the *Sphagnum* genus^42,43^. As such, we suggest that there is the opportunity and need for further investigation of many more *Sphagnum* species in the detail we present here. Using two model species, we have identified key thresholds in two *Sphagnum* species in their response to drought (Fig. 5), which is vital to understanding how peatlands will behave under future climate scenarios. These thresholds differ between species according to their ecological niche and reflects their relative ability to recover from periods without rain, which in turn will have serious consequences for carbon (C) cycling and storage in peatland ecosystems.

### C flux response to drought

Both species of *Sphagnum* were initially robust to removal of water, with NEE similar between the controls and drought microcosms after the first week in both species. It was after this that differences emerged between the two species (Fig. 1). Even though the effect of drought on C flux was not immediately apparent after one week, damage was incurred in both species during that time, and after rewetting NEE subsequently declined and was less than in the controls. There was a divergence in recovery between the two species: NEE recovered more quickly in the hummock forming *S. palustre* than in the lawn species *S. squarrosum. S. palustre* could also withstand a longer drought period. Whereas *S. palustre* droughted for one week recovered to its pre-drought NEE by week five, *S. squarrosum* did not recover to those levels even given ten weeks of recovery time. It took a drought of two weeks to take *S. palustre* to a point that it did not recover predrought NEE. This variation in *Sphagnum* drought recovery reflects previous studies, where the hummock species *S. fuscum* made a full recovery after 11 days of drought, but two other *Sphagnum* species, lawn species *S. majus* and the *S. balticum* did not^44^. Likewise, though *S. capilliforum* (hummock) also demonstrated the ability to recover from a short drought of one week after being rewetted for 20 days^45^it failed to recover from a longer drought of 80 days, even after a 30 day rewetting period^38^. Five *Sphagnum* spp., including the hummock species *S. papillosum* and *S. cuspidatum*, failed to recover at all from a relatively short drought period (12 days)^46^but this may have been due to the short rewetting time of just 12 h; both species showed signs of recovering from a longer drought of 23 days, when given 16 days to recover, but only recovered to ca. 25% of the predrought C uptake^47^.

The response of both species to rewetting was an initial burst of CO_2_ release, which became progressively larger with a longer drought period. This effect has been seen before^46,47^ and it has been suggested that it may be due to heterotrophic respiration of the *Sphagnum* surface microbiome, rather than the *Sphagnum* itself^38^. What is clear in our study is that the rewetting CO_2_ burst is entirely down to respiration, rather than a balance between respiration and GPP, though we cannot determine whether that respiration is hetero- or autotrophic. Over the course of three weeks after rewetting, GPP slowly increased, while respiration declined toward zero in both species, allowing some recovery of the C sink of *Sphagnum*.

Another important aspect of C cycling in *Sphagnum* is methane (CH_4_) flux. Initially, both species exhibited a sink for CH_4_, a property of *Sphagnum* peatlands considered important for reducing net CH_4_ emissions^48^. The magnitude of this initial sink was in line with the higher end of the range reported in *Sphagnum* from around the world^49^but quickly shut down over the course of the experiment, independent of any drought treatment applied. The prevalence of CH_4_ oxidation in *Sphagnum* is attributed to the community of microorganisms which associate with this moss; we suggest that as opposed to the high concentrations of CH_4_ which would usually be found in a peatland, the microcosms in this study were exposed to ambient CH_4_ concentrations, resulting in substrate limitation for this community and therefore a switch in microbial resource strategy.

### *Sphagnum* water content and relation to C flux

There was a clear difference in the moisture contents of the two *Sphagnum* species, with the hummock species, *S. palustre*, having a higher moisture content throughout than the lawn species, *S. squarrosum*. This supports the hypothesis that the hummock species, occupying microhabitats further from the water table, is better adapted to water retention. There are contradictory viewpoints in the literature regarding this hypothesis, with some studies arguing that hollow species should be expected to be more drought tolerant^32^while others suggest that by retaining water more efficiently, hummock species are more drought tolerant^47^. To further support this hypothesis, our analysis indicates that the optimum water content of *S. palustre* (ca. 12 g g^− 1^) was considerably lower than that of *S. squarrosum* (ca. 18 g g^− 1^). This allowed *S. palustre* to maintain its photosynthetic function for longer after the onset of drought, a period which was extended further by the fact that its moisture content was higher to start with. Both species exhibited a reduction in NEE either side of their optimum moisture content, though rather than a symmetrical relationship^38^there was a clear asymmetry as reported elsewhere^46,50^. This optimum moisture content appears to also represent a threshold: for both species in our study, the week during which biomass moisture content dropped below the optimum was also the length of drought period from which NEE did not recover to the level of the controls. As such, our study suggests that rather than drought length, it is drought severity (how dry *Sphagnum* biomass becomes) which will determine the consequences for peatlands. The results should therefore be interpreted within the context of moisture thresholds rather than drought length. Strikingly, there was a strong hysteresis in the relationship between moisture content and NEE, seen in both *Sphagnum* species. After rewetting, moisture content had no influence on NEE. There appears to be very little, if any, reports of such hysteresis in the literature, which may well have considerable implications for remote sensing approaches used to assessing peatland status (see below). Knowledge of a species-specific moisture threshold for NEE will be extremely valuable for predicting how the C dynamics of peatlands will respond to future drought scenarios.

### Hyperspectral reflectance after drought

Bleaching is a well-known drought response of *Sphagnum*^51^and the biomass of both species in this study visibly changed as it dried, becoming much paler over time. This was supported by changes seen in the spectra from the different drought treatments. Multiple vegetation indices presented here showed progressive declines with increasing drought length, and also exhibited strong relationships with NEE. As with moisture content, there appeared to be thresholds of drought length beyond which each species did not recover. Although NDVI is used widely^52,53^structure independent pigment index (SIPI) was a better predictor of NEE in both species. In *S. squarrosum*, the strength of the relationship (slope of regression line) between SIPI and NEE declined sharply below ca. 0.84, suggesting rapid loss of photosynthetic capacity even at high levels of ‘greenness’^54^. In contrast, *S. palustre* had a much less-well pronounced change in the relationship at a SIPI value of ca. 0.72. In *S. palustre*, biomass rewetted after one week of drought demonstrated SIPI similar to controls, whereas SIPI did not recover in any of the droughted *S. squarrosum* to the same level of non-droughted biomass. A similar pattern was seen in the other indices calculated from the spectra. These relationships point to irreversible damage incurred by the *Sphagnum* species due to drought, and could be used in remote sensing to indicate monitor potential drought damage to NEE over time. However, our results indicate that caution must be applied when using spectral indices to track changes in NEE as the relationship between moisture and C flux breaks down once the species-specific drought moisture threshold is exceeded.

We have demonstrated a clear difference in the response of two *Sphagnum* species to drought. The hummock species *S. palustre* was characterised by both a greater capacity for drought avoidance (retaining moisture for longer), drought tolerance (sustaining GPP at lower moisture content) and recovery (regaining pre-drought levels of GPP). We have identified species-specific thresholds of desiccation which are indicators of long-term suppression of carbon uptake.

It is likely that peatlands with hummock species such as *S. palustre* will remain a C sink for longer under future climate scenarios. *Sphagnum* is a keystone species for peatland restoration^17^ and the identification of species drought specific thresholds for carbon uptake has important implications in the selection of species mix for restoration work. With northern peatlands facing up to 82% more desiccation events, it is vital that the most drought tolerant species be used when reintroduction of *Sphagnum* species is undertaken^11^.

The breakdown of the relationship between moisture and C flux is also important: the use of water indices in remote sensing to infer GPP will not be valid if the *Sphagnum* has undergone drought beyond the thresholds identified here. However, the recovery curves we have identified following rewetting, if used with information regarding previous moisture status might be used as a proxy for C flux and will also be a powerful tool for modelling future GPP following periods of drought. The thresholds that we have identified provide crucial new information which can guide modelling, policy and monitoring of this important global C sink.

### Methods

#### Experimental design

The drought experiment was conducted between August and November 2024 at the University of Manchester’s Firs Environmental Research Station, in Fallowfield, Manchester, UK. *S. palustre* and *S. squarrosum* were chosen as the two study species. *Sphagnum* was sourced from a commercial micropropagation company (Beadamoss, Leicestershire, UK) who developed a technique to produce plugs used for peatland restoration. An individual plug consists of 100–200 individual *Sphagnum* plants to be planted in bare peat during restoration programmes.

For this study, one plug was used for each microcosm. Fifty microcosms of each *Sphagnum* species (total of 100) were constructed using cylindrical Lock n Lock containers (volume of 700 cm^3^) which were used because they transmit light effectively (88.65 ± 1.59% of external photosynthetically active radiation (PAR)) and have airtight lids which were adapted to allow GHG flux measurements to be taken easily. Starting biomass of each microcosm was ascertained by weighing each plug and drying a sub sample (1 to 2 plants) at 60 °C to calculate the fresh weight to dry weight ratio (fw/dw). The microcosms were assigned to an experimental block based on starting biomass: total biomass for each species was ranked, and 1–10 for each species were placed in block 1, 2–20 in block two etc. so that there were five blocks consisting of twenty microcosms (ten of each species).

After transplanting the plugs into the microcosms, 100 ml of rainwater (NO_3_ ~ 0 ppm, SO_4_ < 200 mg L^− 1^) collected on site was added to each. A two-week acclimation period then followed, where microcosms were regularly monitored and topped up to the 100 ml level using the same rainwater. During this time microcosms were left open to the air, outside, on metal shelving with a south facing orientation. At the end of the two-week acclimation period, drought treatments were then assigned within each block at random. Drought consisted of the removal of all standing water from a microcosm and a complete withdrawal of any precipitation. Microcosms were replaced on the outdoor shelving and a rain canopy was placed over the top to prevent ingress of rainwater. The canopy consisted of polytunnel material stretched over a timber frame, which allowed transmission of 61.43 ± 1.68% of ambient PAR. The sides were open to the air to maintain conditions as close to ambient as possible. The environmental conditions (air temperature, relative humidity, solar radiation) were recorded hourly at the site throughout the experimental period (Fig. S7). At the end of each week, one droughted treatment was rewetted by adding rainwater to the 100 ml level, and subsequently watered along with the controls for the remainder of the study (Table S1). This created a design where in week 1, there were five controls and 45 droughted microcosms for each species; in week 2, this altered to five controls, five rewetted and 40 droughted microcosms. Subsequently the number of droughted microcosms reducing by a further five each week. Water levels in controls were monitored every few days at the beginning of the study, and it was established that weekly watering was sufficient to maintain inundation in the control and rewetted microcosms.

#### *Sphagnum* moisture content

Every week, the moisture content of the biomass was measured in each microcosm, by removing 1–2 individual capitula, gently blotting the excess water with tissue paper and weighing the fresh biomass. The biomass was then dried in an oven at 60 °C until constant weight and the fw/dw calculated.

#### Carbon fluxes

Weekly exchange of carbon dioxide (CO_2_) and methane (CH_4_) from each microcosm was measured using a cavity ring down laser greenhouse gas analyser (LGR UGGA, Los Gatos, USA). Briefly, standing water was removed and the microcosm was sealed using an adapted Lock and Lock lid, with a sampling tube and a return tube to allow headspace air to be circulated between the analyser and the microcosm. A small (< 1 mm diameter) hole was drilled in the lid to allow equalisation of pressure between the headspace and the atmosphere. Microcosms were sealed for one minute and flux was calculated as the rate of change in concentration over time, using linear regressions, and corrected for dry biomass, so that fluxes were expressed as moles of gas per gram of *Sphagnum* per second, with negative fluxes indicating net uptake and positive fluxes net release. All measurements were conducted under controlled light and temperature conditions in a growth chamber at 20 °C and 200 µmol m^− 2^ s^− 1^ photosynthetically active radiation (PAR), light level which will sustain more than 90% of the maximum potential photosynthesis of both *Sphagnum* species^55^. Partway through the study, in response to the emerging data, it was decided it was necessary to partition NEE into dark respiration (Rd) and photosynthesis (gross primary productivity, GPP). To achieve this, following a measurement of NEE, the microcosm was vented to the air to allow re-equilibration with ambient CO_2_ and then re-closed. The microcosm was darkened by completely covering with aluminium foil and Rd was determined from a second flux measurement taken over the course of one minute. GPP was then calculated by subtracting Rd from NEE.

#### Hyperspectral reflectance

At the end of the study, the hyperspectral reflectance of the vegetation was measured in all 100 microcosms. A field spectrometer (SVC HR-1024i, Spectra Vista Corporation, NY, USA) with a 4° field of view was mounted on a boom, 57 cm above the target at 90° angle, allowing a measurement area of ca. 12.5 cm^2^ around the centre of the microcosm. Measurements were taken in a windowless laboratory, with the only light source a 1 kW halogen lamp used to illuminate the target. Each microcosm was measured twice, one measurement immediately after the other, rotating the target by 180° to account for shading by the sides of the microcosm. Reflectance was averaged over the two measurements for each microcosm. Reflectance from the vegetation was calculated as the target radiance divided by the radiance from a calibrated Spectralon reference panel, which was measured prior to each microcosm.

#### Vegetation indices

Several vegetation indices were calculated from the hyperspectral reflectance data based on the reflectance at given wavelengths (ρ (nm)) (Table S1).

### Statistical analyses

Linear mixed effects models were used to test *Sphagnum* moisture content, NEE and respiration, and CH_4_ fluxes, for differences between fixed effects of species, drought treatment and time, with experimental block as a random effect. For the models, *n* = 100.

To investigate the recovery of CO_2_ fluxes over time, non-linear regression was used with the model:

NEE = initial flux * exp^(A * week_number)^ + final flux.

The optimum moisture content of *Sphagnum* for NEE was calculated using segmented linear regression^56^. The same approach was used for identifying the breakpoints in the association between SIPI and NEE in both *Sphagnum* species (Fig. 6a and b). Tests of association between vegetation indices and CO_2_ flux were conducted using linear regression. All statistical analyses were performed and figures produced using SAS 9.4 (SAS Institute, Cary, NC USA). For all statistical tests, a P-value of 0.05 and lower was used to identify significant result.

## Electronic supplementary material

Below is the link to the electronic supplementary material.

Supplementary Material 1

## Data Availability

The data that support the findings of this study are available from the corresponding author upon reasonable request.
