# Supplementary material for: Recovery of Sphagnum from drought is controlled by species-specific moisture thresholds
Source: Sci Rep. 2025 Jul 1;15:22167. doi: 10.1038/s41598-025-05348-8 (PMC12216555; doi:10.1038/s41598-025-05348-8)
Supplement: Supplementary file 1 — Supplementary Material 1 [file 41598_2025_5348_MOESM1_ESM.docx]

**Title:** Recovery of *Sphagnum* from drought is controlled by species-specific moisture thresholds

**Authors:**

Ben Keane^1,2^ ben.keane@manchester.ac.uk 0000-0001-7614-8018

Emma L. Shuttleworth^1^ emma.shuttleworth@manchester.ac.uk 0000-0003-0661-1366

Martin G. Evans^1,3^ martin.g.evans@durham.ac.uk 0000-0003-2300-1033

Jonathan P. Ritson^1^ jonny.ritson@manchester.ac.uk 0000-0001-5753-1863

Angela Harris^1^ angela.harris@manchester.ac.uk https://orcid.org/0000-0002-2184-0274

Adam Johnston^1,4^ adam.johnston@manchester.ac.uk 0000-0002-6788-0407

Danielle M. Alderson^1^ danielle.alderson@manchester.ac.uk 0000-0001-8072-9923

Gareth D. Clay^1^ gareth.clay@manchester.ac.uk 0000-0002-8477-2774

1: School of Environment, Education and Development, The University of Manchester, Oxford Rd, Manchester, M13 9PL, UK

2: Department of Environment and Geography, University of York, Yor, UK YO10 5NG

3: Department of Geography, Durham University, Durham, DH1 3LE

4: JBA Consulting, 1 Broughton Park, Old Lane North, Broughton, Skipton, North Yorkshire, BD23 3FD

**Key words:** *Sphagnum*, drought, recovery, methane, carbon, peatland

**Supplementary Information**

Table S1 Schedule of experimental plan. There were five replicates of each species-drought treatment combination. Shaded areas indicate treatments maintained at inundation, shaded and hashed areas indicate (rewetted) recovery period.

| **Species** | **Drought treatment** | **Week** | | | | | | | | | |  |
| --- | --- | --- | --- | --- | --- | --- | --- | --- | --- | --- | --- | --- |
|  |  | **0** | **1** | **2** | **3** | **4** | **5** | **6** | **7** | **9** | **11** | |
| *S. palustre* | C | 5 | 5 | 5 | 5 | 5 | 5 | 5 | 5 | 5 | 5 | |
|  | D1 | 5 | 5 | 5 | 5 | 5 | 5 | 5 | 5 | 5 | 5 | |
|  | D2 | 5 | 5 | 5 | 5 | 5 | 5 | 5 | 5 | 5 | 5 | |
|  | D3 | 5 | 5 | 5 | 5 | 5 | 5 | 5 | 5 | 5 | 5 | |
|  | D4 | 5 | 5 | 5 | 5 | 5 | 5 | 5 | 5 | 5 | 5 | |
|  | D5 | 5 | 5 | 5 | 5 | 5 | 5 | 5 | 5 | 5 | 5 | |
|  | D6 | 5 | 5 | 5 | 5 | 5 | 5 | 5 | 5 | 5 | 5 | |
|  | D7 | 5 | 5 | 5 | 5 | 5 | 5 | 5 | 5 | 5 | 5 | |
|  | D9 | 5 | 5 | 5 | 5 | 5 | 5 | 5 | 5 | 5 | 5 | |
|  | Drought | 5 | 5 | 5 | 5 | 5 | 5 | 5 | 5 | 5 | 5 | |
| *S. squarrosum* | C | 5 | 5 | 5 | 5 | 5 | 5 | 5 | 5 | 5 | 5 | |
|  | D1 | 5 | 5 | 5 | 5 | 5 | 5 | 5 | 5 | 5 | 5 | |
|  | D2 | 5 | 5 | 5 | 5 | 5 | 5 | 5 | 5 | 5 | 5 | |
|  | D3 | 5 | 5 | 5 | 5 | 5 | 5 | 5 | 5 | 5 | 5 | |
|  | D4 | 5 | 5 | 5 | 5 | 5 | 5 | 5 | 5 | 5 | 5 | |
|  | D5 | 5 | 5 | 5 | 5 | 5 | 5 | 5 | 5 | 5 | 5 | |
|  | D6 | 5 | 5 | 5 | 5 | 5 | 5 | 5 | 5 | 5 | 5 | |
|  | D7 | 5 | 5 | 5 | 5 | 5 | 5 | 5 | 5 | 5 | 5 | |
|  | D9 | 5 | 5 | 5 | 5 | 5 | 5 | 5 | 5 | 5 | 5 | |
|  | Drought | 5 | 5 | 5 | 5 | 5 | 5 | 5 | 5 | 5 | 5 | |


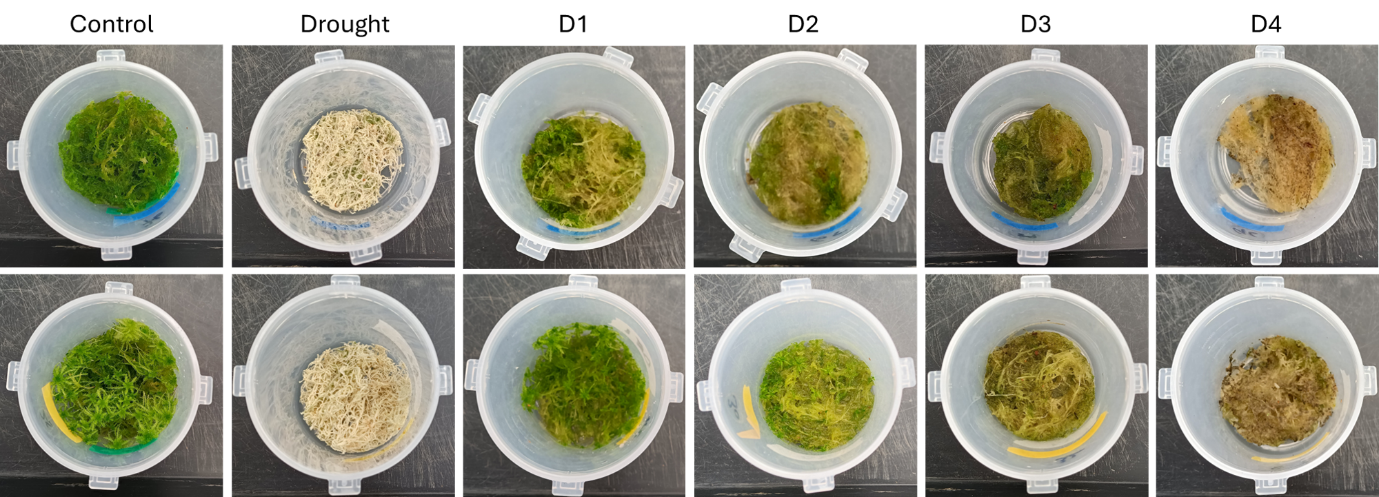


Figure S1 example microcosms after five weeks of the drought experiment. Two *Sphagnum* species, *S. palustre* (top row) and *S. squarrosum* (bottom row) maintained at full inundation (control), removal of all water (drought) and rewetted in recovery in sequential weeks (e.g. D2 rewetted after two weeks of drought, D4 after four weeks).

Table S2 Vegetation indices calculated from hyperspectral data at various wavelengths (ρ (nm)).

| Spectral Index | Wavelength / formula | Property detected | Reference |
| --- | --- | --- | --- |
| Normalised difference vegetation index (NDVI) | ρ 842 – ρ 665 / ρ 842 + ρ 665 | Broadband greenness | Rouse *et al.* (1974) ^58^ |
| Carotenoid reflectance index (CRI) | (1/ ρ 510) – (1/ ρ 550) | Leaf pigment (carotenoid) | Gitelson *et al.* (2002) ^59^ |
| Plant senescence reflectance index (PSRI): | ρ 680 - ρ 500 / ρ 750 | Dry / senescent carbon | Merzlyak *et al.* (1999) ^60^ |
| Cellulose absorbance index (CAI): | 0.5 x (ρ 2000 + ρ 2200) – ρ 2100 | Dry / senescent carbon | Daughtry *et al.* (1996) ^61^ |
| Anthocyanin reflectance index (ARI): | 1/ ρ 550 – 1/ ρ 700 | Leaf pigment (anthocyanin) | Gitelson *et al.* (2001) ^62^ |
| Photochemical reflectance index (PRI): | ρ 531 – ρ 570 / ρ 531 + ρ 570 | Light use efficiency | Gamon *et al.* (1992) ^63^ |
| Structure insensitive pigment index (SIPI): | ρ 800 – ρ 445 / ρ 800 – ρ 680 | Light use efficiency | Penuelas *et al.* (1995) ^55^ |
| Red edge position (REP): | 700 + 40 x (ρ REP – ρ 700 / ρ 740 – ρ 700) | Narrowband greenness | Guyot & Baret 1988^64^; Curran *et al.* (1995) ^65^ |





Figure S2 Methane (CH_4_) flux measured from microcosms of two *Sphagnum* species (*S. palustre* (top panel) and *S. squarrosum* (bottom panel)). Microcosms were kept inundated (control), droughted (drought) or droughted then rewetted after a number of weeks (e.g. D1= rewetted after 1 week drought). Negative values of NEE indicate net uptake of CH_4_. Vertical dashed lines indicate timing of drought and recovery periods. Data shown are means for each treatment (n= 5 ± 1SE, with the exception of drought, where initial n= 45 and declined by 5 each week).


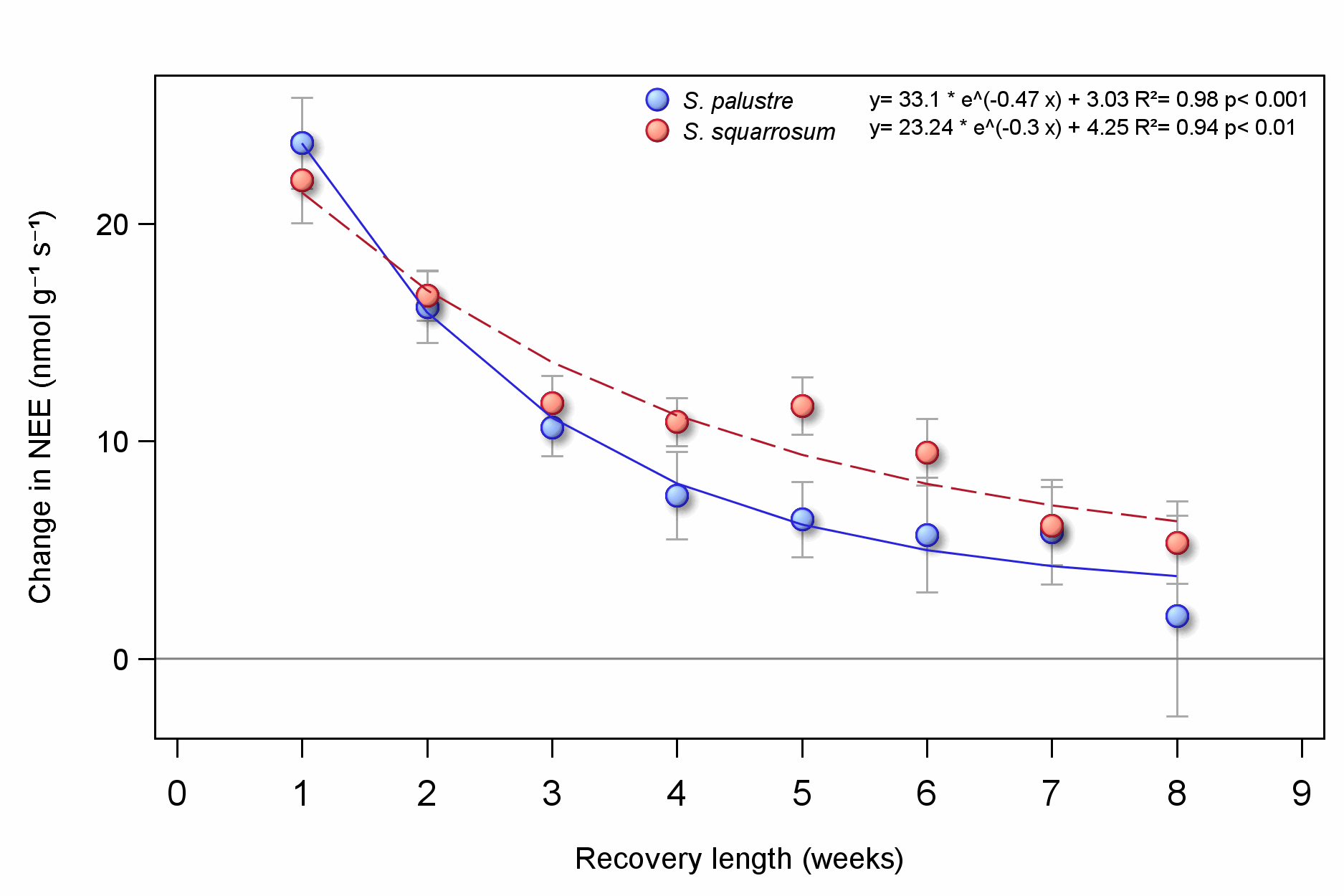


Figure S3 Variation in relative CO_2_ flux (difference in NEE between recovering microcosms and control microcosms) from two *Sphagnum* species (*S. palustre*, blue symbols and regression line, and *S. squarrosum*, red symbols and regression line) following rewetting after drought. The zero line represents the rate of NEE in controls; fluxes above this (positive fluxes) indicate increased loss of CO_2_ from *Sphagnum* and fluxes below (negative fluxes) indicate increase uptake of CO_2_. Fluxes are means ± 1SD and the regression line fitted is a negative exponential, formulae in the top right of the panel.

Table S3 Properties of recovery curves from *Sphagnum* species after rewetting (Fig. 4)

| **Species** | **Drought length (weeks)** | **Final relative NEE (nmol g^-1^ s^-1^)** |
| --- | --- | --- |
| *S. palustre* | 1 | 0.22 |
| *S. palustre* | 3 | 9.64 |
| *S. palustre* | 4 | 8.64 |
| *S. palustre* | 5 | 8.72 |
| *S. palustre* | 6 | 8.02 |
| *S. palustre* | 7 | 15.15 |
| *S. squarrosum* | 1 | 7.26 |
| *S. squarrosum* | 3 | 10.67 |
| *S. squarrosum* | 4 | 11.41 |
| *S. squarrosum* | 5 | 11.89 |
| *S. squarrosum* | 6 | 11.6 |
| *S. squarrosum* | 7 | . |





Figure S4 Gross primary productivity (GPP), net ecosystem exchange (NEE) and dark respiration (Rd) from microcosms of two species of *Sphagnum*: controls were kept inundated and recovery denotes *Sphagnum* which had undergone 6 weeks of drought, followed by one week of rewetting. Data shown are means for each treatment (n= 5 ± 1SE).

**

**

Figure S5 Hyperspectral data from two *Sphagnum* spp. (*S. palustre*, top panel, and *S. squarrosum*, bottom panel), under different levels of drought and recovery (Control, constant drought (Drought) and rewetted after one week drought (D1) up to nine weeks drought (D9)). Data shown are means ± 1SE, n = 5.

**

**

Figure S6 Eight different vegetation indices (Normalised differential vegetation index (NDVI), carotenoid reflectance index (CRI), cellulose absorbance index (CAI), plant senescence reflectance index (PSRI)), photochemical reflectance index (PRI), anthocyanin reflectance index (ARI), structure independent pigment index (SIPI) and red edge position (REP), calculated from the spectra of two *Sphagnum* spp. (*S. palustre*, blue, and *S. squarrosum*, red), under different levels of drought and recovery (Control, constant drought (Drought) and rewetted after one week drought (D1) up to nine weeks drought (D9).

**

**

Figure S7 Environmental variables recorded from a weather station onsite during the experimental period.
